# MnO_2_–graphene nanosheets wrapped mesoporous carbon/sulfur composite for lithium–sulfur batteries

**DOI:** 10.1098/rsos.171824

**Published:** 2018-02-07

**Authors:** Zhengzheng Li

**Affiliations:** Automobile Steel Research Institute, R&D Center, BaoWu Group Corporation, Shanghai 201900, People's Republic of China

**Keywords:** MnO_2_ nanosheets, lithium–sulfur batteries, carbon/sulfur composite

## Abstract

MnO_2_–graphene nanosheets wrapped mesoporous carbon/sulfur (MGN@MC/S) composite is successfully synthesized derived from metal–organic frameworks and investigated as cathode for lithium-ion batteries. Used as cathode, MGN@MC/S composite possesses electronic conductivity network for redox electron transfer and strong chemical bonding to lithium polysulfides, which enables low capacity loss to be achieved. MGN@MC/S cathodes exhibit high reversible capacity of 1475 mA h g^−1^ at 0.1 C and an ultra-low capacity fading of 0.042% per cycle at 1 C over 450 cycles.

## 1. Introduction

The development of cost-efficient and energy-efficient battery systems that meet future electric vehicle demands is urgently needed [1]. Lithium–sulfur (Li-S) batteries are regarded as a promising candidate due to the fact that the low-cost elemental sulfur provides three to five times the state-of-art Li-ion battery theoretical specific capacity (1675 mA h g^−1^) [2–5]. Moreover, sulfur is abundant and environmentally friendly, which makes it attractive for large-scale commercial applications. Despite these advantages, many basic obstacles still restrict the practical application of Li-S batteries, including (i) the intrinsically insulative nature of sulfur, intermediate polysulfides and Li_2_S that result in low utilization of active materials and rate capability, (ii) severe volume change (approx. 80%) during the lithiation/delithiation processes, leading to the destruction of the electrode materials and (iii) the dissolution and shuttling effect of intermediate lithium polysulfides (Li_2_S*_x_*, 4 ≤ *x* ≤ 8) that give rise to low coulombic efficiency, poor cycling stability and self-discharge [6–9].

In response to these challenges, great efforts have been devoted to enhance the electrical conductivity of cathodes and suppress the loss of soluble polysulfide intermediates during cycling. The use of conductive carbon scaffolds (porous carbon, carbon nanotubes and graphene) to obtain a nanostructured composite S cathode is an efficient and effective way for Li-S batteries [10–15]. They can serve as the host scaffold to accommodate the S/Li_2_S and demonstrate significant improvement for the cathode capacity. However, a carbon framework is less effective in trapping polysulfides because of the weak interaction between the non-polar carbons and the polar polysulfides [12,13]. Recently, metal oxides have been paid attention due to their chemisorption effect towards polysulfide species, resulting in considerable gains in coulombic efficiency and cycling stability. For example, some metal oxides (such as TiO_2_ [6], Ti_4_O_7_ [16] and MnO_2_ [17–20]) as traps for the polysulfides have proven to be highly effective at chemically adsorbing of lithium polysulfides and improving the long-cycle ability of Li-S batteries. However, these materials are less conductive than carbon materials, which leads inevitably to compromises in the rate capability and even the specific capacity [19]. Therefore, it may be a good alternative to realize the complementary advantages of metal oxides and carbon materials, in which the carbon materials provide high electric conductivity, while the polar metal oxides create reactive sites for immobilization of lithium polysulfides. However, it is still a great challenge to design the hybrid structures of polar metal oxides and highly conductive carbon materials.

Considering the above discussion, here, we designed and synthesized a MnO_2_–graphene nanosheets coated mesoporous carbon/sulfur (MGN@MC/S) composite, in which the conductive mesoporous carbon (MC) network greatly reduces the resistance of electron transport and mass diffusion during the discharge and charge processes, thus giving rise to high rate capabilities; MnO_2_ nanosheets provide strong binding sites for polysulfide intermediates and promote stable redox activity over the whole lifetime of the cathode material. As a result, MGN@MC/S composite exhibits superior electrochemical performance in terms of reversible specific capacity (1115 mA h g^−1^ at 0.1 C), rate capability (551 mA h g^−1^ at 1 C) and cycling stability (0.05% capacity decay per cycle over more than 500 cycles at 1 C).

## 2. Material and methods

### 2.1. Synthesis of MnO_2_–graphene nanosheets wrapped mesoporous carbon/sulfur composite

#### 2.1.1. Chemicals

2-Aminoterephthalic acid (99%), aluminium chloride hexahydrate (99%), sulfur (98%), NaHSO_3_ (98%) were obtained from Sigma-Aldrich Chemical Co. and used as received.

#### 2.1.2. Synthesis of Al-MIL-101-NH_2_

The synthesis of Al-MIL-101-NH_2_ was based on a modified method as reported in [21]. The typical synthesis of Al-MIL-101-NH_2_ was carried out as follows: 544 mg (3 mmol) of 2-aminoterephthalic acid was dissolved in 120 ml of dimethylformamide (DMF) and heated to 110°C in an oil bath. In total, 1448 mg (6 mmol) of AlCl_3 _· 6H_2_O in seven equal portions was added in the above solution every 15 min. After that, the reaction went on at 110°C for 3 h under stirring and then kept standing for an additional 16 h. After being cooled down to room temperature, the yellow solid was isolated by filtration, washed with 100 ml of DMF and ethanol three times, and then further purified by treatment in ethanol at 80°C for 24 h. The yellow solid was finally dried for 12 h at 200°C under vacuum for further use.

#### 2.1.3. Preparation of mesoporous carbon

The Al-MIL-101-NH_2_ samples were loaded into a ceramic boat and placed into a tube furnace under an argon flow, heated from room temperature to 800°C in 80 min, and then kept at 800°C for 6 h and cooled down to room temperature. The pyrolysed black materials were treated with an HF (23 wt%) solution for 12 h, followed by filtration and dried at 80°C for 12 h to afford MC.

#### 2.1.4. Preparation of mesoporous carbon/sulfur

Sulfur and MC were mixed according to a mass ratio of 7 : 3, yielding a black mixture, which was sealed in a glass container and heated at 155°C for 24 h under vacuum condition.

#### 2.1.5. Preparation of the MnO_2_ nanosheets

MnO_2_ nanosheets were synthesized by a one-step facile method using graphene oxide (GO) as template [17]. Briefly, 20 ml of GO suspension (1 mg ml^−1^) was dispersed in 80 ml distilled water by sonication. In total, 160 mg of KMnO_4_ was added into the GO suspension and stirred at room temperature for 30 min. The mixture was transferred into a thermostatic oven at 80°C for 24 h. The resulting material was washed with distilled water and dried at 80°C for 12 h to afford MnO_2_ nanosheets.

#### 2.1.6. Fabrication of MGN@MC/S

On total, 20 mg MnO_2_ nanosheets and 100 mg MC/S were homogeneously dispersed into 20 ml of as-fabricated GO suspension (1 mg ml^−1^) by mild ultrasonication for 60 min. Next, 0.15 g NaHSO_3_ was added as the assistant reducing agent, and the mixture was ultrasonically dispersed in an ice bath for 30 min. Then, the mixture was placed in a 40 ml sealed bottle and maintained at 80°C for 6 h. After that, the bottle was naturally cooled to room temperature and the as-formed hydrogel was poured out and dialysed with 1 l distilled water for 24 h. Last, the hydrogel was frozen at −50°C for 3 h and then freeze-dried for 3 days to obtain the MGN@MC/S composite.

### 2.2. Materials characterization

The content of sulfur was analysed using a thermogravimetric analyser (Diamond PE) under an Ar atmosphere at a heating rate of 10°C min^−1^ from room temperature to 600°C, with a gas flow-rate of 40 ml min^−1^. Scanning electron microscope (SEM) observation was carried out on a FEI Quanta 650 SEM operated at 20 kV. Scanning transmission electron microscopy (STEM) was conducted with a Hitachi S-5500 SEM, and energy-dispersive X-ray spectroscopy was applied for collecting elemental signals and mapping. Transmission electron microscopy (TEM) and high-resolution TEM images were recorded with a JEOL-2100 instrument. Powder X-ray diffraction (XRD) was conducted on a Bruker D8 Advance X-ray diffractometer using Cu K*α* radiation at a scanning rate of 4° min^−1^ in the 2*θ* range from 10° to 70°. Specific surface area, pore volume and pore-size distribution were determined by the Brunauer–Emmett–Teller (BET) method on a Micromeritics ASAP 2020 instrument.

### 2.3. Electrochemical measurements

To prepare the working electrodes, as-prepared composite was mixed with acetylene black and poly(vinyl difluoride) in a weight ratio of 8 : 1 : 1 in *N*-methyl pyrrolidone solution. The slurry was pasted onto a carbon paper and then dried in a vacuum oven at 60°C overnight. The active material density of each cell was determined to be 1.5–2.0 mg cm^−2^. CR2025-type coin cells were fabricated by sandwiching a porous polypropylene separator between the film disc of MGN@MC/S and a lithium metal foil in a high-purity argon-filled glove box. A measure of 1 M LiN(CF_3_SO_2_)_2_ in a mixed solvent of 1,3-dioxolane and dimethyl ether (1 : 1 by volume) with 2wt% anhydrous lithium nitrate were used as the electrolyte. Cyclic voltammetry (CV) measurements were conducted with a Zahner Zennium Electrochemical Workstation in the potential range 0.01–3.0 V at a scan rate of 0.1 mV s^−1^.

## 3. Results and discussion

Metal–organic frameworks (MOFs) as a new class of organic–inorganic hybrid materials have received much attention in the last decade due to their great potential for clean energy applications [22–25]. As a family of porous materials, the employment of MOFs as precursors to prepare porous carbon materials shows great potential for applications in energy and environmental fields. Among them, Al-MIL-101-NH_2_ with a high carbon content and large surface area is a good candidate to synthesize porous carbon directly upon carbonization. The synthesis of Al-MIL-101-NH_2_ was performed by a solvothermal route in DMF solvent. The obtained Al-MIL-101-NH_2_ was demonstrated by powder XRD (electronic supplementary material, figure S1 [26]). Direct carbonization of the precursor and removal of Al_2_O_3_ with an HF solution affords MC materials. The XRD patterns reveal two broad peaks located at around 25° and 44° that are assigned to the graphitic structure (figure 2*a*) [21].

**Figure 1.**
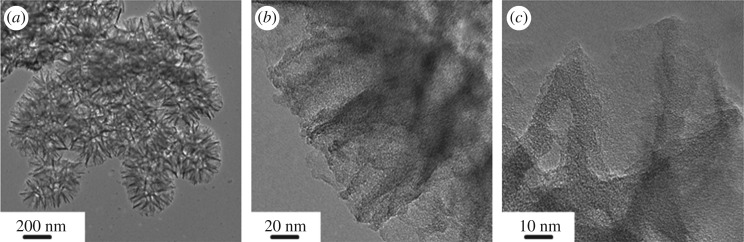
TEM images of the fabricated MC composite.

The porous structures and nanotopography of the MC are evaluated by nitrogen adsorption–desorption and TEM analysis. The calculated BET surface area of the carbon material is 1328 m^2^ g^−1^ with a pore volume of 0.7 cm^3^ g^−1^, and the pore-size distribution lies in the range of 3–4 nm (electronic supplementary material, figure S2(a), (b) [26]). The TEM and highly resolved TEM images proved the existence of nanopores over the entire material surface (figure 1). The nanoporous structure of MC is anticipated to play important roles in achieving high sulfur loading and alleviating the shuttle effect.

**Figure 2.**
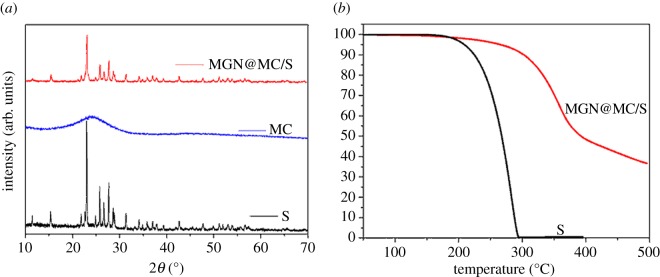
(*a*) XRD patterns of MC, S and MGN@MC/S. (*b*) Thermogravimetric analysis of S and MGN@MC/S.

The MC/S is prepared by a melt-diffusion method at 155°C. To diminish the dissolution of polysulfides at the surface of the MC, we coat a layer of MGN on the surface of the MC/S electrode. After the hydrothermal and freeze-drying process, MGN@MC/S composite is obtained. The XRD results show a monoclinic phase sulfur (JCPDS-01-080-1098) [17] in the MGN@MC/S composite. The sulfur content in MGN@MC/S is calculated to be 64 wt% (figure 2*b* and electronic supplementary material, figure S6) by thermogravimetric analysis. The HCS elemental analysis reveals that the MGN@MC/S composites consist of approximately 15.5 wt % MnO_2_ nanosheets. MnO_2_ nanosheets have been demonstrated to chemisorb polysulfide mediators, which can prevent the dissolution of long-chain Li_2_S*_x_* (*x* = 4–8) into the organic electrolyte, leading to a long cycling stability [17–20].

**Figure 3.**
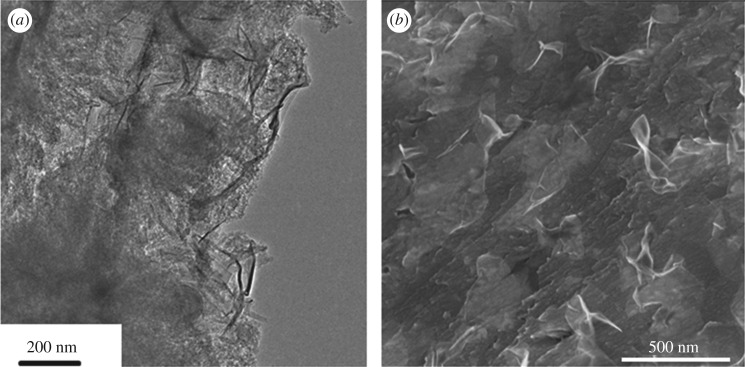
(*a*) TEM and (*b*) SEM images of the MGN@MC/S composite.

**Figure 4.**
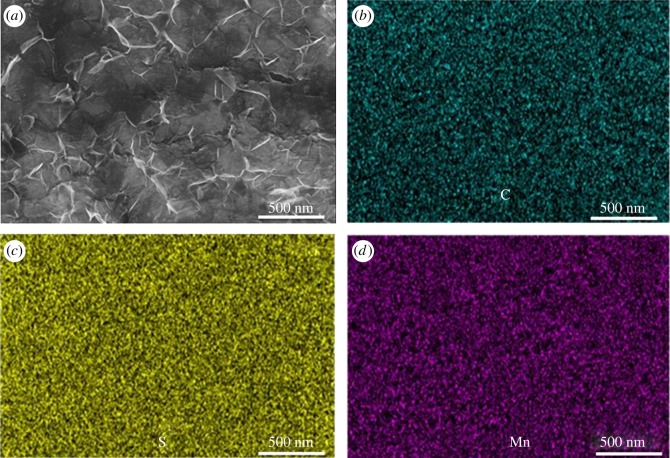
(*a*) SEM image of the MGN@MC/S composite. (*b*–*d*) Elemental mapping for C, S, Mn from the same area shown in the image of (*a*).

TEM images reveal that the whole MC/S electrode is wrapped by MnO_2_ and graphene sheets with a thickness of 4 nm (figure 3*a* and electronic supplementary material, figures S3 and S4 [26]). SEM images of the MGN@MC/S composite show that a layer of MnO_2_ and graphene sheets cover the surface of the MC/S electrode (figure 3*b* and electronic supplementary material, figure S5 [26]). STEM images reveal that no large sulfur particles are observed in the MGN@MC/S composite (figure 4*a*). The energy dispersive X-ray spectroscopy mapping clearly reveals the uniform distributions of C, S and Mn elements, suggesting the homogeneous distribution of S on the MGN@C framework and Mn element dispersed around sulfur (figure 4*b–d*). These results demonstrate that the MGN@C nanostructure effectively accommodates the sulfur inside the carbon framework.

**Figure 5.**
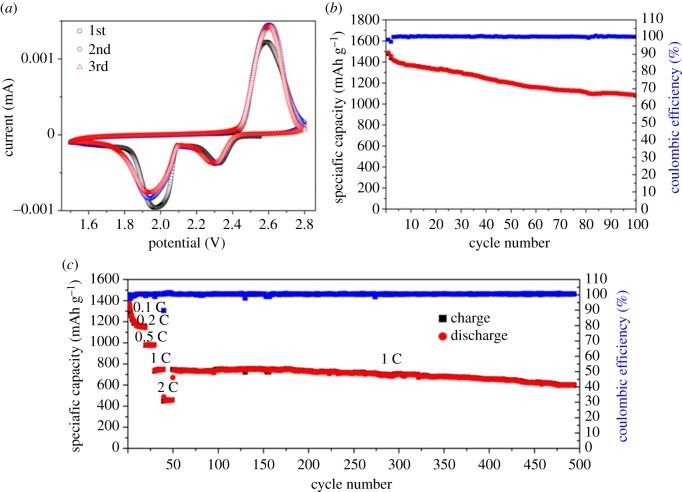
(*a*) CV profile of the MGN@MC/S electrode at a scan rate of 0.1 mV s^−1^; (*b*) cycle performance of the MGN@MC/S electrode at 0.1 C; (*c*) the rate performance and cycle performance of the MGN@MC/S electrode.

Electrochemical Li-storage performance of the MGN@MC/S composite has been evaluated by using CR2025 coin cells. The electrochemical reaction mechanism of the MGN@MC/S cathode was studied by CV at a scan rate of 0.1 mV s^−1^ in the potential range of 1.5–2.8 V (versus Li/Li^+^), as shown in figure 5*a*. A pair of pronounced redox peaks and one oxidation peak were observed. The two reduction peaks at potentials of 2.3 and 2.0 V are attributed to reduction of the cyclic sulfur molecules to the long-chain lithium polysulfides (Li_2_S*_n_*, 4 < *n* < 8), and further reduction to lower-order polysulfides (Li_2_S_2_ and Li_2_S) [27,28]. An anodic peak at approximately 2.45 V is associated with the conversion of the lower-order polysulfides to polysulfides and sulfur molecules. From the second cycle to the fifth cycle, no remarkable changes can be observed for both the reduction and oxidation peaks, indicating high reversible cycling stability of the MGN@MC/S cathode.

The discharge and charge behaviour of the MGN@MC/S electrode was evaluated at 0.1 C rates (1 C = 1675 mA g^−1^) in the voltage window of 1.9–2.6 V (figure 5*b*). The pure MC and pure graphene–MnO_2_ seem to have nearly no capacity at 1.9–2.6 V with a current above 0.1 C (167.5 mA g^−1^) as shown in electronic supplementary material, figure S7. This suggests that the capacity of MGN@MC/S is almost totally contributed by sulfur particles in the MGN@MC/S composite. This phenomenon is consistent with the reported literature [29,30]. The material exhibited an initial discharge capacity of 1475 mA h g^−1^, corresponding to 88% sulfur utilization. The high sulfur utilization indicated the highly dispersed nanosized sulfur particles in the MGN@MC/S composite. After 100 cycles, a high discharge capacity of 1088 mA h g^−1^ was retained with a coulombic efficiency of 99%, which corresponds to 74% of the capacity retention. Figure 5*c* shows the rate performance of the MGN@MC/S composite cathodes at various rates. With increasing current rate, the discharge capacity gradually decreased, for example, at 0.1 C (1362 mA h g^−1^), 0.2 C (1190 mA h g^−1^), 0.5 C (983 mA h g^−1^), 1 C (748 mA h g^−1^) and 2 C (452 mA h g^−1^), indicating a good rate capability. In addition, the coulombic efficiency of the MGN@MC/S electrode was nearly 100% at all the current rates. These results indicate that a little shuttling effect was achieved due to the strong binding sites of MnO_2_ nanosheets for polysulfide intermediates [17,18]. The MGN@MC/S electrode also displays remarkably long cycling performance charged at 1 C rate. When the rate returns to 1 C, the specific capacity of 742 mA h g^−1^ recovers, implying high stability of the electrode materials. A high specific capacity of 600 mA h g^−1^ is retained after 450 cycles. The capacity retention is calculated to be 80%, corresponding to a very low capacity decay of 0.042% per cycle. The superior cycling stability of the MGN@MC/S electrode is attributed to the conductive MC network and the strong binding sites of MnO_2_ nanosheets for polysulfide intermediates. The conductive MC network greatly reduces the resistance of rapid electron transport and mass diffusion; MnO_2_ nanosheets provide strong chemical binding to polysulfides. Therefore, the MGN@MC/S composite could efficiently immobilize sulfur and polysulfide, leading to maximum interaction between sulfur and carbon host and minimal dissolution of lithium polysulfide intermediates.

## 4. Conclusion

MGN@MC/S composite is successfully synthesized derived from MOFs and investigated as cathode for lithium-ion batteries. The MGN@MC/S composite possesses electronic conductivity network for redox electron transfer and strong chemical binding to lithium polysulfides, which enables low capacity loss to be achieved. Used as cathodes of Li-S batteries, the MGN@MC/S cathodes exhibit high reversible capacity of 1475 mAh g^−1^ at 0.1 C and an ultra-low capacity fading of 0.042% per cycle at 1 C over 450 cycles. This is attributed to the reduced resistance of electron and ion transport in the conductive MC network and strong chemical affinity to polysulfides provided by MnO_2_ nanosheets. These results indicate that Li-S batteries with high energy densities and long-cycle stability could be achieved by properly designing sulfur cathodes.

## Supplementary Material

XRD patterns;BET isotherms;TEM images;TEM images;SEM images

## Data accessibility

Data have been deposited with Dryad and can be accessed at http://dx.doi.org/10.5061/dryad.dv22q [26].

## References

[1] Ji X, Evers S, Black R, Nazar LF 2011 Stabilizing lithium–sulphur cathodes using polysulphide reservoirs. Nat. Commun. 2, 325 (doi:10.1038/ncomms1293) 21610728 10.1038/ncomms1293

[2] Cuisinier M, Cabelguen P-E, Adams BD, Garsuch A, Balasubramanian M, Nazar LF 2014 Unique behaviour of nonsolvents for polysulphides in lithium–sulphur batteries. Energy Environ. Sci. 7, 2697–2705. (doi:10.1039/c4ee00372a)

[3] Qie L, Manthiram A 2015 A facile layer-by-layer approach for high-areal-capacity sulfur cathodes. Adv. Mater. 27, 1694–1700. (doi:10.1002/adma.201405689) 25605465 10.1002/adma.201405689

[4] Xiao Z, Yang Z, Wang L, Nie H, Zhong M, Lai Q, Xu X, Zhang L, Huang S 2015 A lightweight TiO_2_/graphene interlayer, applied as a highly effective polysulfide absorbent for fast, long-life lithium-sulfur batteries. Adv. Mater. 27, 2891–2898. (doi:10.1002/adma.201405637) 25820906 10.1002/adma.201405637

[5] Li X, Liang J, Zhang K, Hou Z, Zhang W, Zhu Y, Qian Y 2015 Amorphous S-rich S1−xSex/C (x ≤ 0.1) composites promise better lithium–sulfur batteries in a carbonate-based electrolyte. Energy Environ. Sci. 8, 3181–3186. (doi:10.1039/c5ee01470k)

[6] Wei Seh Z, Li W, Cha JJ, Zheng G, Yang Y, McDowell MT, Hsu P-C, Cui Y 2013 Sulphur–TiO_2_ yolk–shell nanoarchitecture with internal void space for long-cycle lithium–sulphur batteries. Nat. Commun. 4, 1331 (doi:10.1038/ncomms2327) 23299881 10.1038/ncomms2327

[7] Tao X et al. 2016 Balancing surface adsorption and diffusion of lithium-polysulfides on nonconductive oxides for lithium–sulfur battery design. Nat. Commun. 7, 11203 (doi:10.1038/ncomms11203) 27046216 10.1038/ncomms11203 PMC4822044

[8] Zheng G, Zhang Q, Cha JJ, Yang Y, Li W, Seh ZW, Cui Y 2013 Amphiphilic surface modification of hollow carbon nanofibers for improved cycle life of lithium sulfur batteries. Nano Lett. 13, 1265–1270. (doi:10.1021/nl304795g) 23394300 10.1021/nl304795g

[9] Liang X et al. 2015 Tuning transition metal oxide-sulfur interactions for long life lithium sulfur batteries: the ‘goldilocks’ principle. Adv. Energy Mater. 6, 1501636 (doi:10.1002/aenm.201501636)

[10] Pang Q, Tang J, Huang H, Liang X, Hart C, Tam KC, Nazar LF 2015 A nitrogen and sulfur dual-doped carbon derived from polyrhodanine@cellulose for advanced lithium-sulfur batteries. Adv. Mater. 27, 6021–6028. (doi:10.1002/adma.201502467) 26314378 10.1002/adma.201502467

[11] Li G, Sun J, Hou W, Jiang S, Huang Y, Geng J 2016 Three-dimensional porous carbon composites containing high sulfur nanoparticle content for high-performance lithium–sulfur batteries. Nat. Commun. 7, 10601 (doi:10.1038/ncomms10601) 26830732 10.1038/ncomms10601 PMC4740444

[12] Wang Z, Dong Y, Li H, Zhao Z, Bin Wu H, Hao C, Liu S, Qiu J, Lou XW (David) 2014 Enhancing lithium–sulphur battery performance by strongly binding the discharge products on amino-functionalized reduced graphene oxide. Nat. Commun. 5, 5002 (doi:10.1038/ncomms6002) 25255431 10.1038/ncomms6002

[13] Li H, Yang X, Wang X, Liu M, Ye F, Wang J, Qiu Y, Li W, Zhang Y 2015 Dense integration of graphene and sulfur through the soft approach for compact lithium/sulfur battery cathode. Nano Energy 12, 468–475. (doi:10.1016/j.nanoen.2015.01.007)

[14] Xu F, Tang Z, Huang S, Chen L, Liang Y, Mai W, Zhong H, Fu R, Wu D 2015 Facile synthesis of ultrahigh-surface-area hollow carbon nanospheres for enhanced adsorption and energy storage. Nat. Commun. 6, 7221 (doi:10.1038/ncomms8221) 26072734 10.1038/ncomms8221 PMC4490369

[15] Ding Y-L, Kopold P, Hahn K, van Aken PA, Maier J, Yu Y 2016 Lithium-sulfur batteries: facile solid-state growth of 3D well-interconnected nitrogen-rich carbon nanotube-graphene hybrid architectures for lithium-sulfur batteries (Adv. Funct. Mater. 7/2016). Adv. Funct. Mater. 26, 1144 (doi:10.1002/adfm.201670046)

[16] Pang Q, Kundu D, Cuisinier M, Nazar LF 2014 Surface-enhanced redox chemistry of polysulphides on a metallic and polar host for lithium-sulphur batteries. Nat. Commun. 5, 4759 (doi:10.1038/ncomms5759) 25154399 10.1038/ncomms5759

[17] Liang X, Hart C, Pang Q, Garsuch A, Weiss T, Nazar LF 2015 A highly efficient polysulfide mediator for lithium–sulfur batteries. Nat. Commun. 6, 5682 (doi:10.1038/ncomms6682) 25562485 10.1038/ncomms6682

[18] Liang X, Nazar LF 2016 In situ reactive assembly of scalable core–shell sulfur–MnO_2_ composite cathodes. ACS Nano 10, 4192–4198. (doi:10.1021/acsnano.5b07458) 26910648 10.1021/acsnano.5b07458

[19] Li Z, Zhang J, Lou XWD 2015 Frontispiz: hollow carbon nanofibers filled with MnO_2_ nanosheets as efficient sulfur hosts for lithium-sulfur batteries. Angew. Chem. 127, 13 078–13 082. (doi:10.1002/ange.201584461) 10.1002/anie.201506972 26349817

[20] Wang X, Li G, Li J, Zhang Y, Wook A, Yu A, Chen Z 2016 Structural and chemical synergistic encapsulation of polysulfides enables ultralong-life lithium–sulfur batteries. Energy Environ. Sci. 9, 2533–2538. (doi:10.1039/c6ee00194g)

[21] Sun J-K, Xu Q 2014 From metal–organic framework to carbon: toward controlled hierarchical pore structures via a double-template approach. Chem. Commun. 50, 13 502–13 505. (doi:10.1039/c4cc06212d) 10.1039/c4cc06212d 25236386

[22] Wu HB, Xia BY, Yu L, Yu X-Y, Lou XW (David) 2015 Porous molybdenum carbide nano-octahedrons synthesized via confined carburization in metal-organic frameworks for efficient hydrogen production. Nat. Commun. 6, 6512 (doi:10.1038/ncomms7512) 25758159 10.1038/ncomms7512 PMC4382699

[23] Zhong H, Wang J, Zhang Y, Xu W, Xing W, Xu D, Zhang Y, Zhang X 2014 ZIF-8 derived graphene-based nitrogen-doped porous carbon sheets as highly efficient and durable oxygen reduction electrocatalysts. Angew. Chem. Int. Ed. 53, 14 235–14 239. (doi:10.1002/anie.201408990) 10.1002/anie.201408990 25331053

[24] Sorribas S, Zornoza B, Téllez C, Coronas J 2012 Ordered mesoporous silica–(ZIF-8) core–shell spheres. Chem. Commun. 48, 9388 (doi:10.1039/c2cc34893d) 10.1039/c2cc34893d 22892418

[25] Tang J, Wu S, Wang T, Gong H, Zhang H, Alshehri SM, Ahamad T, Zhou H, Yamauchi Y 2016 Cage-type highly graphitic porous carbon–Co_3_O_4_ polyhedron as the cathode of lithium–oxygen batteries. ACS Appl. Mater. Interfaces 8, 2796–2804. (doi:10.1021/acsami.5b11252) 26788868 10.1021/acsami.5b11252

[26] Li Z 2018 Data from: MnO_2_–graphene nanosheets wrapped mesoporous carbon/sulfur composite for lithium–sulfur batteries Dryad Digital Repository. (doi:10.5061/dryad.dv22q) 10.1098/rsos.171824 PMC5830778 29515889

[27] Liang Z, Zheng G, Li W, Seh ZW, Yao H, Yan K, Kong D, Cui Y 2014 Sulfur cathodes with hydrogen reduced titanium dioxide inverse opal structure. ACS Nano 8, 5249–5256. (doi:10.1021/nn501308m) 24766547 10.1021/nn501308m

[28] Yuan Z, Peng H-J, Huang J-Q, Liu X-Y, Wang D-W, Cheng X-B, Zhang Q 2014 Hierarchical free-standing carbon-nanotube paper electrodes with ultrahigh sulfur-loading for lithium-sulfur batteries. Adv. Funct. Mater. 24, 6105–6112. (doi:10.1002/adfm.201401501)

[29] Lai H, Li J, Chen Z, Huang Z 2012 Carbon nanohorns as a high-performance carrier for MnO_2_ anode in lithium-ion batteries. ACS Appl. Mater. Interfaces 4, 2325–2328. (doi:10.1021/am300378w) 22545767 10.1021/am300378w

[30] Lou F, Zhou H, Tran TD, Melandsø Buan ME, Vullum-Bruer F, Rønning M, Walmsley JC, Chen D 2014 Coaxial carbon/metal oxide/aligned carbon nanotube arrays as high-performance anodes for lithium ion batteries. ChemSusChem 7, 1335–1346. (doi:10.1002/cssc.201300461) 24578068 10.1002/cssc.201300461

