# Supplementary material for: MnO2–graphene nanosheets wrapped mesoporous carbon/sulfur composite for lithium–sulfur batteries
Source: R Soc Open Sci. 2018 Feb 7;5(2):171824. doi: 10.1098/rsos.171824 (PMC5830778; doi:10.1098/rsos.171824)
Supplement: XRD patterns;BET isotherms;TEM images;TEM images;SEM images [file rsos171824supp1.doc]

**Royal Society Open Science**

**Supplementary Material**

# MnO_2_-Graphene Nanosheets wrapped Mesoporous Carbon/Sulfur Composite for Lithium-Sulfur Batteries

Zhengzheng Li*

Automobile Steel Research centre, Research Institute Of Baosteel Group

655Fujin Road, Baoshan District, Shanghai,201900,China

E-mail: Lizhengzheng@baosteel.com

**
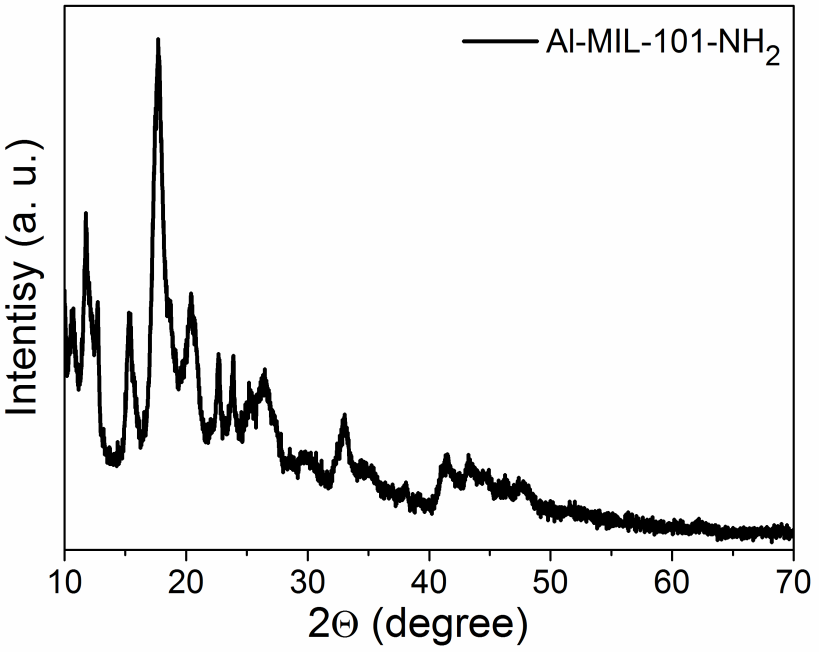
**

**Fig. S1** XRD patterns of as-synthesized of Al-MIL-101-NH_2_.

The obtained Al-MIL-101-NH2 was demonstrated by powder X-ray diffraction (PXRD) (Fig. S1).

**Synthesis of Al-MIL-101-NH_2_**

The synthesis of Al-MIL-101-NH_2_ was based on a modified method as reported in Ref.^21^ The typical synthesis of Al-MIL-101-NH_2_ was carried out as follows: 544 mg (3 mmol) of 2-aminoterephthalic acid was dissolved in 120 mL of DMF and heated to 110 °C in an oil bath. 1448 mg (6 mmol) of AlCl_3_·6H_2_O in 7 equal portions was added in the above solution every 15 min. After that, the reaction went on at 110 °C for 3 h under stirring and then kept standing for an additional 16 h. After being cooled down to room temperature, the yellow solid was isolated by filtration, washed with 100 mL of DMF and ethanol for three times, and then further purified by treatment in ethanol at 80 ºC for 24 h. The yellow solid was finally dried for 12 h at 200 °C under vacuum for further use.


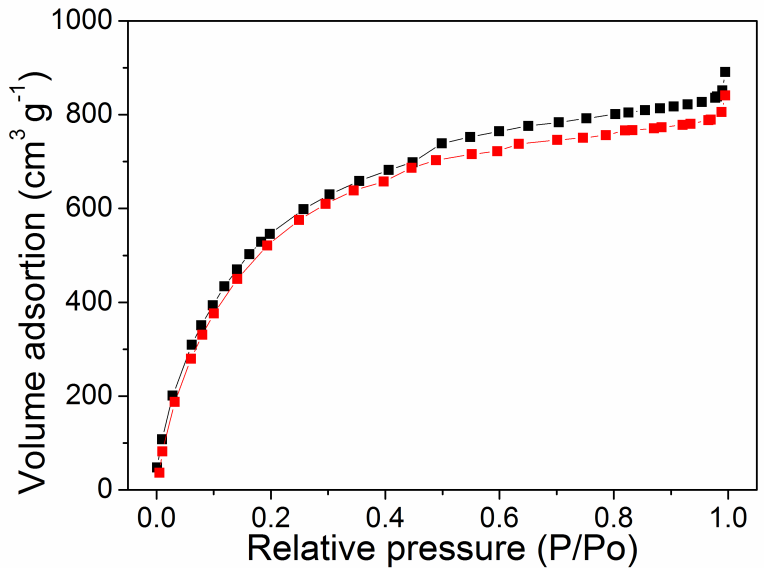


**Fig. S2a** BET nitrogen adsorption-desorption isotherms of MC.


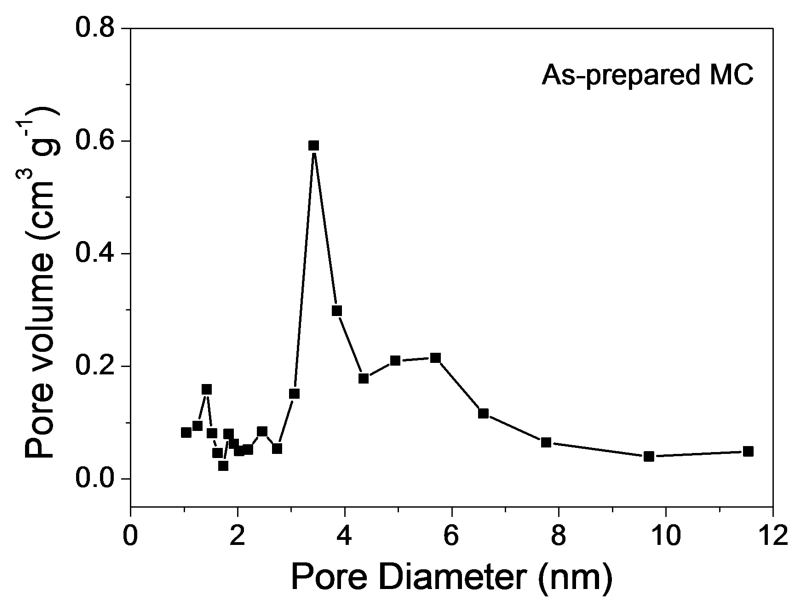


**Fig. S2b** Pore size distributions of as-prepared MC.

The porous structures and nanotopography of the MC are evaluated by nitrogen adsorption-desorption and TEM analysis. The calculated BET surface area of the carbon material is 1328 m2 g-1 with a pore volume of 0.7 cm3 g-1 (Fig. S2a), and the pore-size distribution lies in 3-4 nm range (Fig. S2b [28]).

**Preparation of mesoporous carbon (MC)**

The Al-MIL-101-NH2 samples were loaded into a ceramic boat and placed into a tube furnace under an argon flow, heated from room temperature to 800 °C in 80 min, and then kept at 800 °C for 6 h and cooled down to room temperature. The pyrolyzed black materials were treated with a HF (23 wt%) solution for 12 h, followed by filtration and dried at 80 °C for 12 h to afford mesoporous carbon.


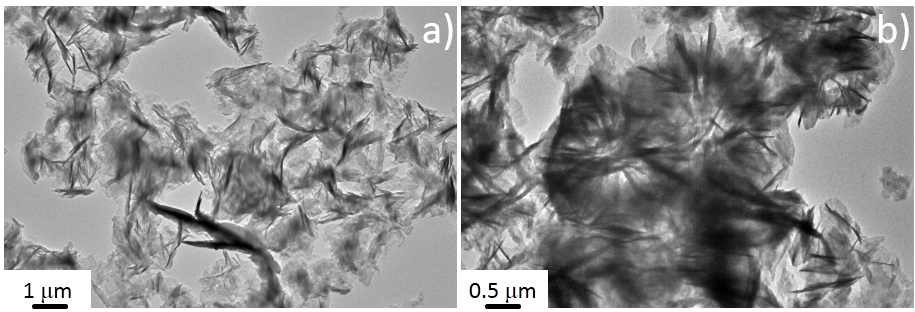


**Fig. S3** a, b) TEM images of the MnO_2_ nanosheets.


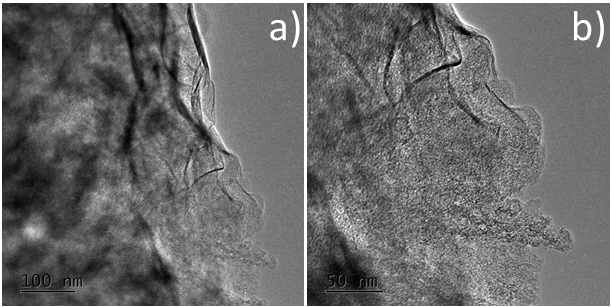


**Fig. S4** TEM images of the fabrication of MGN@MC/S.

TEM images reveal that the whole MC/S electrode is wrapped by MnO2 and graphene sheets with a thickness of 4 nm. (Fig. S3, S4).

**Preparation of the MnO_2_ nanosheets**

MnO_2_ nanosheets were synthesized by a one-step facile method using GO as template. ^17^ Briefly, 20 mL of GO suspension (1 mg mL^-1^) were dispersed in 80 ml distilled water by sonication. 160 mg of KMnO_4_ was added into the GO suspension and stirred at room temperature for 30 min. The mixture was transferred into a thermostatic oven at 80 ^o^C for 24 h. The resulting material was washed with distilled water and dried at 80 °C for 12 h to afford MnO_2_ nanosheets.


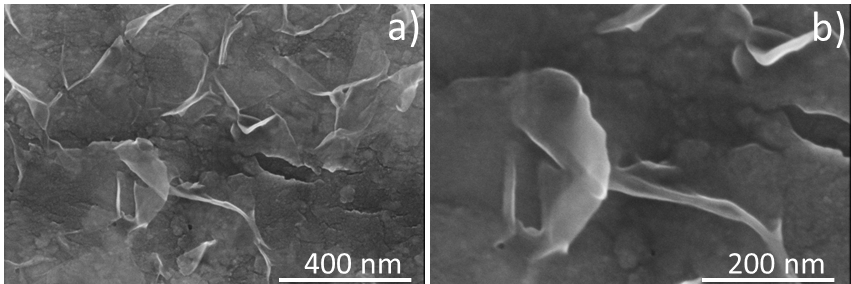


**Fig. S5** SEM images of the fabrication of MGN@MC/S.

Scanning electron microscope (SEM) images of the MGN@MC/S composite show that a layer of MnO2 and graphene sheets cover on the surface of the MC/S electrode ( Fig. S5).

**Fabrication of MGN@MC/S**

20 mg MnO2 nanosheets and 100 mg MC/S were homogeneously dispersed into 20 mL of as-fabricated GO suspension (1 mg mL^-1^) by mild ultrasonication for 60 min. Next, 0.15 g NaHSO_3_ was added as the assistant reducing agent, and the mixture was ultrasonically dispersed in an ice bath for 30 min. Then, the mixture was placed in a 40 mL sealed bottle and maintained at 80 ^o^C for 6 h. After that, the bottle was naturally cooled to room temperature and the as-formed hydrogel was poured out and dialyzed with 1 L distilled water for 24 h. Last, the hydrogel was frozen at -50 ^o^C for 3 h and then freeze-dried for 3 days to obtain the MGN@MC/S composite.


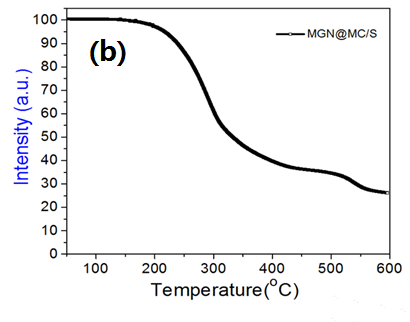


**Fig. S6 TGA of MGN@MC/S**

It is really true as Reviewer suggested that a tail of loss of weight over 500 oC is found comparing to Fig. 2b after we repeated the TGA measurement of MGN@MC/S, as shown in Fig. S6. This tail is derived from the reduction reaction of carbon and MnO2. Therefore, the sulfur content in MGN@MC/S is calculated to be 64 wt % (Fig. 2b and Fig. S6) by the Thermo gravimetric analysis (TGA).

**Fig. S7 the electrochemical data of pure mesoporous carbon (MC) and pure graphene-MnO2 (MGN)**

The pure mesoporous carbon and pure graphene-MnO2 seems to be nearly no capacity at 1.9-2.6 V with a current above 0.1 C (167.5 mA/g) as shown in Fig S7. This suggests the capacity of MGN@MC/S is almost totally contributed by sulfur particles in the MGN@MC/S composite. This phenomenon is consistence with the reported literatures (ACS Appl. Mater. Interfaces, 2012, 4 (5), 2325-2328, ChemSusChem, 2013, 7: 1335-1346).
